# Improved Chromatographic Alignment of GC-IMS Data Using Spectral Information

**DOI:** 10.1021/acsmeasuresciau.6c00271

**Published:** 2026-10-02

**Authors:** Luis Fernández, Gema Guedes de la Cruz, Eduardo Caballero, Eva Martín-López, Albert Vidal, Javier Alonso-Valdesueiro, Josep Gumà, Raquel Cumeras, Antonio Pardo, Santiago Marco

**Affiliations:** † Department of Electronics and Biomedical Engineering, 16724 Universitat de Barcelona, 08028 Barcelona, Spain; ‡ Institute for Bioengineering of Catalonia (IBEC), The Barcelona Institute of Science and Technology, 08028 Barcelona, Spain; § Institut d’Oncologia de la Catalunya Sud (IOCS), Hospital Universitari Sant Joan de Reus, Facultat de Medicina i Ciències de la Salut, Universitat Rovira i Virgili, 43204 Reus, Spain; ∥ Institut de Recerca Biomèdica Catalunya Sud (IRBCatSud), 43204 Reus, Spain

**Keywords:** GC-IMS, chromatographic alignment, peak matching, peak correspondence, correspondence metrics, correlation optimized warping

## Abstract

Gas chromatography-ion mobility spectrometry (GC-IMS) provides two-dimensional separation of volatile compounds, but instrumental variability can produce shifts and nonlinear distortions that hinder comparison among measurements. This study presents a chromatographic alignment workflow that uses spectral information from the drift-time dimension to support peak correspondence. The method combines injection point correction and parametric time warping along the retention-time axis with multiplicative correction of the drift-time axis. Peaks are then matched using information from both dimensions, and their retention positions are used as internal landmarks for a final cubic spline refinement without requiring standards specifically for alignment. The workflow was evaluated using 134 repeated measurements of a pooled urine sample and compared with correlation optimized warping (COW) applied directly and after different preprocessing stages. Conventional metrics based on correlation, Euclidean similarity, simplicity, and peak factor indicated comparable global performance for the proposed method and the best COW workflows. However, a correspondence-based assessment showed that high profile similarity and low positional dispersion did not necessarily imply preservation of the same peak correspondences. Direct COW recovered 45.2% of the reference correspondences, whereas recovery increased to 85.0% after injection point correction and to 89.9% after injection point correction plus parametric time warping. These results highlight the importance of combining profile-based metrics with measures of peak correspondence and show how the orthogonal drift-time dimension can provide additional constraints for chromatographic alignment in GC-IMS data.

## Graphical abstract

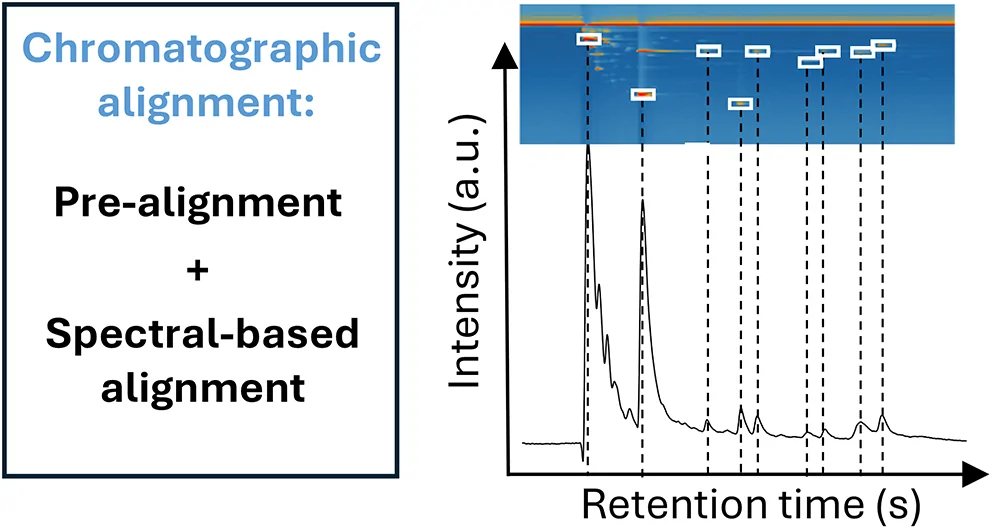

## 1 Introduction

Metabolomics is the systematic analysis of metabolites in biological systems to investigate changes associated with physiological, pathological, or environmental conditions.^1^ Its applications include biomarker discovery, personalized medicine, toxicological assessment, agriculture and nutrition, and microbial and industrial biotechnology.^2^ Metabolomic studies often involve large numbers of biological samples containing complex mixtures of metabolites whose concentrations may vary among individuals and over time.^3–6^ Reliable characterization therefore depends on appropriate sample collection, transport, storage, and control of potential confounding factors.^7,8^

The analytical techniques most commonly used in metabolomics include nuclear magnetic resonance, liquid chromatography coupled with mass spectrometry, and gas chromatography coupled with mass spectrometry.^9^ Gas chromatography coupled with ion mobility spectrometry, or GC-IMS, has also gained relevance in metabolomic studies.^10–13^ In GC-IMS, volatile compounds are first separated in a chromatographic column according to their interaction with the stationary phase. After elution, they are ionized and further separated according to their mobility through a drift tube.^14,15^ Each signal is therefore characterized by both retention time and drift time. Compared with other coupled techniques such as LC-MS and GC-MS, GC-IMS is relatively cost-efficient, although it generally provides lower selectivity.^16,17^

GC-IMS measurements can be represented as two-dimensional signal maps in which compounds appear as peaks defined by retention time and drift time. Reliable comparison among samples requires adequate noise reduction, baseline correction, resolution of overlapping signals, and consistent peak correspondence across measurements. Digital filters such as moving average and Savitzky–Golay filters can reduce noise,^18^ whereas asymmetric least squares and related methods can be used for baseline correction.^19,20^ Wavelet-based methods can assist with peak detection,^21,22^ while Multivariate Curve Resolution with Alternating Least Squares and Parallel Factor Analysis can help resolve overlapping components.^23,24^ Once peaks have been detected, clustering methods may be used to group corresponding signals across samples.^25,26^

Small changes in instrumental and experimental conditions can cause shifts and nonlinear distortions along both measurement axes.^27^ These variations are often more pronounced along the chromatographic axis and can hinder the correspondence of peaks across samples. Several one-dimensional profiles can be derived from a GC-IMS measurement to facilitate correction. The reversed reactant-ion peak chromatogram summarizes changes in reactant-ion peak intensity along the retention-time axis, whereas the total ion spectrum summarizes the signal along the drift-time axis. Variation in drift time can be corrected by aligning the reactant-ion peak in the total ion spectrum of each sample with that of a reference measurement.^28^

Several methods have been developed for chromatographic alignment. Parametric time warping estimates a polynomial transformation that minimizes the difference between a sample chromatogram and a reference chromatogram, whereas correlation optimized warping divides the chromatographic axis into segments and aligns each segment by maximizing its correlation with the corresponding region of the reference.^29,30^ Both methods have subsequently been applied to the chromatographic axis of GC-IMS data^31,32^

Piecewise correction strategies have also been proposed to account for changes in the displacement along the chromatographic axis. Pierce et al. described a general alignment method based on local corrections in different chromatographic regions. Prasad et al. later applied a similar piecewise strategy to pyrolysis gas chromatography coupled with differential mobility spectrometry and evaluated its effect on classification.^33,34^

Previous work has also shown that global and local correction methods can be combined sequentially. Halvorsen et al. combined retention-time locking with correlation optimized warping and found that the combined strategy performed better than either method used separately.^35^ This work illustrates the value of combining complementary alignment stages to address different sources of chromatographic variation. Multichannel information has also been incorporated into chromatographic alignment. In GC-MS data, Nadeau et al. estimated retention-time shifts from the total ion current and transferred the resulting correction to individual mass channels.^36^

Despite these advances, alignment methods based solely on the chromatographic profile do not establish whether peaks brought to similar retention positions correspond to the same chemical features. This limitation is relevant because optimization of global similarity or compactness can produce chromatograms that appear to be well-aligned even when some peak correspondences are inconsistent. In addition, global metrics naturally give greater weight to regions with stronger signals and may be less sensitive to errors affecting smaller peaks.

The two separation dimensions of GC-IMS provide an additional source of information for addressing this problem. A chromatographic peak is associated with one or more signals characterized by their positions along the drift-time axis. After initial correction of both axes, retention time and drift time can therefore be considered jointly to support peak correspondence among samples. The retention positions of the matched peaks can then serve as reference points for a final chromatographic correction. This approach differs from the total ion current (TIC) shift function described for GC-MS, in which a retention-time correction is estimated from the summed ion current and subsequently applied to the individual mass channels.^36^ In the present approach, the drift-time dimension is instead used to establish peak correspondence before defining the final chromatographic correction.

The aim of this study was to develop and evaluate a chromatographic alignment workflow that uses the drift-time dimension of GC-IMS to support peak correspondence. The method first applies injection point correction and parametric time warping to reduce the main sources of variation along the retention-time axis. Variation along the drift-time axis is then corrected based on the position of the reactant-ion peak. Peaks are subsequently matched using information from both retention time and drift time, and their retention positions are used as reference points for a final cubic spline correction. This procedure avoids the addition of internal standards specifically for chromatographic alignment. To the best of our knowledge, the use of drift time to support peak correspondence and subsequently guide spline correction of the chromatographic axis has not previously been reported in GC-IMS.

The method was evaluated by using 134 measurements of pooled urine acquired under controlled experimental conditions. Its performance was compared with correlation optimized warping applied both directly and after different initial correction stages. Conventional metrics, including Pearson correlation, similarity, simplicity, and peak factor, were complemented by a quantitative assessment of how well each workflow preserved the peak correspondences supported by the drift-time dimension. This distinction allowed chromatographic compactness and global signal similarity to be evaluated separately from the consistency of the peak correspondence.

## 2 Materials and Methods

This section outlines GC-IMS analysis, which includes two main stages: sample analysis and data processing. The first stage covers sample collection and the GC-IMS measurement protocol. The second stage focuses on data preprocessing, with particular emphasis on the methods used for chromatographic alignment. Although this study aims to develop a general alignment approach for GC-IMS data, it was applied here to the specific case of urine sample chromatographic alignment for practical validation.

### 2.1 Sample Analysis

Urine samples were collected from 11 healthy volunteers (5 women and 6 men) under a protocol approved by the Ethics Committee of Hospital Universitari Sant Joan de Reus (study approval no. 074/2018). Informed consent was obtained from all participants before sample collection. First-morning fasting urine was collected, excluding the initial and final portions of the stream. Samples were refrigerated for up to 2 h before pooling. The pooled sample was prepared in a laminar flow hood by mixing the individual samples in a sterile glass bottle. While the pool was kept on ice, 134 aliquots of 1.5 mL were prepared in Eppendorf vials, immediately frozen at −80 °C, and stored until analysis. Each vial was subsequently prepared for measurement under sterile conditions. First, 375 mg of sodium chloride was added,^37^ and the vials were kept in an icebox and the vial was kept on ice. After thawing, 200 μL of a 100 ppb 2-decanone solution, used as an internal standard, was added, followed by the urine sample and 86.0 μL of 5 M HCl.^38^ The vials were sealed with crimp caps and kept at room temperature until measurement.

GC-IMS measurements were carried out using a FlavourSpec instrument (G.A.S., Dortmund, Germany). The system was equipped with a nonpolar capillary column (FS-SE-54-CB; 94% methyl, 5% phenyl, and 1% vinylsilicone; 30 m × 0.32 mm ID × 0.25 μm df; CS-Chromatographie Service GmbH, Germany). Ionization was achieved using a tritium source (β-radiation, 300 MBq), and ion separation was performed in a 98 mm drift tube under an electric field of 500 V/cm. After incubation for 15 min at 60 °C, 200 μL of headspace was injected using a PAL-xt autosampler equipped with a syringe heated to 80 °C. Nitrogen (5.0) was used as both carrier gas and drift gas at flow rates of 18 and 200 mL/min, respectively. Volatile compounds were separated in the column at 70 °C and then entered the ionization region. The resulting ions were separated in the drift tube, also maintained at 70 °C, according to their mobility, which depends on factors including the molecular size, shape, and charge. Ions were detected using a Faraday plate that measured ion current over time. Spectra were acquired in positive mode by averaging 12 scans per spectrum, with one spectrum recorded every 32 ms. The grid pulse width was 100 μs, and the sampling rate was 150 kHz. Each measurement lasted 32 min and was followed by a 3 min cleaning phase. A blank consisting of an empty vial was analyzed after each sample to assess carry-over and confirm complete recovery of the reactant-ion peak (RIP). Data were acquired using VOCal software version 0.4.03 (G.A.S., Dortmund, Germany)

Each measurement produced a two-dimensional data matrix defined by retention time and drift time, containing approximately 22.14 million data points. The complete dataset is publicly available through Zenodo at https://zenodo.org/record/18910120.

### 2.2 Data Processing

Data processing comprised a sequence of steps designed to transform the original GC-IMS measurements into aligned chromatograms and to evaluate alignment performance quantitatively. Section 2.2.1 describes the preprocessing of chromatographic and drift-time data. Section 2.2.2 presents the proposed alignment workflow. Section 2.2.3 describes correlation optimized warping, which was used as a comparative method, and Section 2.2.4 presents the metrics used to assess the alignment performance.

Data import, preprocessing, and implementation of the proposed alignment workflow were carried out in R, version 4.4.3, using the GC-IMS package. A detailed description of the package is provided by Oller-Moreno et al.,^31^ and usage examples are available in the package vignettes at https://github.com/sipss/GCIMS/tree/master/vignettes. The preprocessing workflow is summarized in Figure 1.

**Figure 1:**
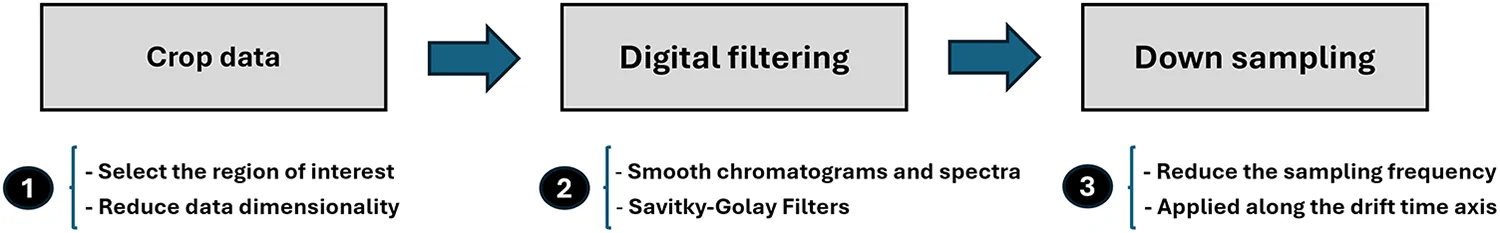
Sequence of preprocessing steps applied to the GC-IMS measurements before chromatographic alignment: cropping of the region of interest, digital filtering, and downsampling of the drift-time axis.

#### 2.2.1 Signal Preprocessing

The GC-IMS data matrices were cropped along both the retention-time and drift-time axes to retain the region containing most of the detected signal. The first 1300 s were retained along the chromatographic axis, and the drift-time range was restricted to 5–17 ms. Cropping reduced the dimensionality to approximately 2.86 million data points per sample. Savitzky–Golay filters were then applied along both axes using a second-order polynomial. The filter window was 3 s along the retention-time axis and 0.14 ms along the drift-time axis. These settings were selected to reduce noise while preserving the peak shape. Finally, the data were downsampled by a factor of 2 along the drift-time axis to reduce the high point density resulting from spectral acquisition. After downsampling, each sample contained approximately 1.43 million data points.

A reference sample was selected for the alignment of the remaining measurements. Ideally, the reference should contain all relevant peaks and be representative of the overall dataset.^39^ For each sample, the maximum cross-correlation with every other sample was calculated and the resulting values were averaged. The sample with the highest mean maximum cross-correlation was selected as the reference. Because cross-correlation allows relative shifts between profiles, this criterion favors samples that are globally representative of the dataset despite differences in the retention time.

#### 2.2.2 Peak Alignment Workflow

Figure 2 summarizes the R-based workflow developed for chromatographic peak alignment. The workflow comprises two main stages. Section 2.2.2.1 describes the prealignment of the retention-time and drift-time axes, whereas Section 2.2.2.2 presents the refinement of chromatographic alignment using peak correspondences supported by the drift-time dimension.

**Figure 2:**
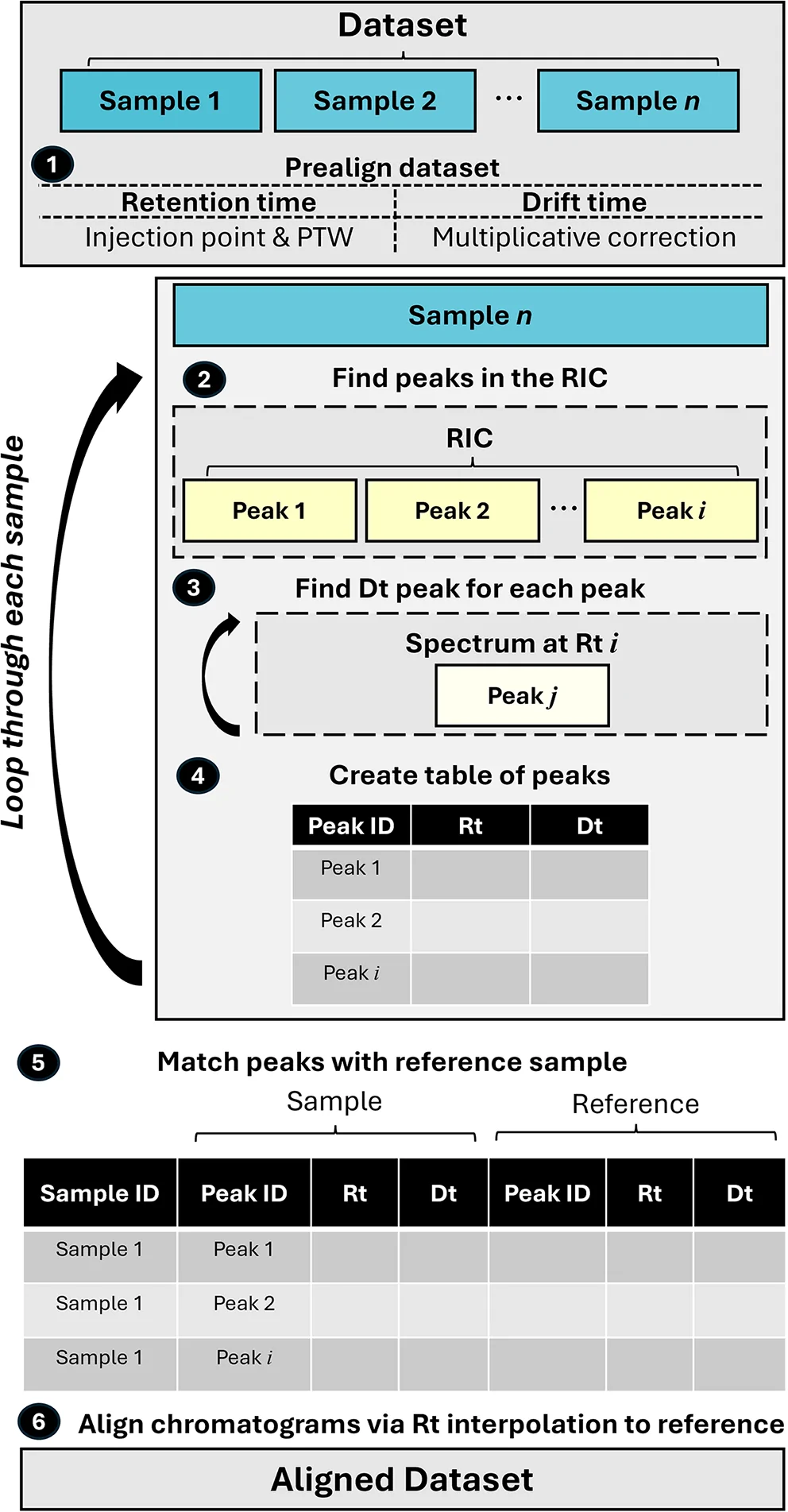
Workflow of the proposed chromatographic alignment method. The procedure comprises prealignment of the retention-time and drift-time axes, peak detection, and correspondence identification using information from both dimensions, and final refinement of the retention-time axis by cubic spline interpolation.

##### 2.2.2.1 Prealignment of the retention-time and Drift-Time Axes

Prealignment was used to correct the main distortions affecting the retention-time and drift-time axes before peak matching. Correction of the retention-time axis comprised two sequential stages: an initial shift correction followed by a nonlinear correction.

The initial shift correction was performed by identifying the injection point in each reversed reactant-ion peak chromatogram (rRIC) and translating the retention-time axis so that the injection point coincided with that of the reference sample. Parametric time warping was then applied to correct the remaining nonlinear variation in retention time. PTW estimates a polynomial warping function that maps the time axis of each sample chromatogram to that of the reference chromatogram. In this study, the polynomial order was restricted to *K* = 2 to favor monotonic transformations and reduce the risk of local oscillations in the corrected chromatograms.^40^ The polynomial coefficients were estimated by approximating the warped chromatogram by using a first-order Taylor expansion and minimizing the squared difference between the warped sample chromatogram and the reference chromatogram.

Variation along the drift-time axis was corrected by using a multiplicative transformation. Temperature and atmospheric pressure affect ion mobility and can therefore alter measured drift times.^41^ The reactant-ion peak was used as a reference because it is consistently present in all sample spectra.^42^ For each sample, the correction factor *K*_c_ was calculated as

1 $$K_{c}=\frac{t_{ref}}{t_{RIP}}$$

where *t*_ref_ is the drift time of the reactant-ion peak in the reference sample and *t*_RIP_ is its drift time in the sample being corrected. The drift-time vector of the sample was multiplied by *K*_c_, thereby aligning the position of the reactant-ion peak with that of the reference.

##### 2.2.2.2 Chromatographic Alignment Based on Spectral Information

Following prealignment, the retention-time axis was refined using peak correspondences supported by the drift-time dimension. Peaks were first detected in the reversed reactant-ion peak chromatograms and then characterized by using the drift-time profiles recorded at their retention positions. Peaks that matched across samples were subsequently used as internal reference points to define the final chromatographic correction relative to the reference sample.

Peaks in the reversed reactant-ion peak chromatograms were detected using the Continuous Wavelet Transform (CWT),^21^ with the Ricker wavelet, also known as the Mexican hat wavelet, as the mother wavelet. Peaks were identified by tracing ridges across scales in the wavelet coefficient space. This approach allows peaks of different widths to be detected because broader peaks generate longer and wider ridges than narrower peaks. A ridge was retained as a peak when its maximum coefficient occurred within the predefined scale range and both its length and signal-to-noise ratio exceeded the selected thresholds.^43^ For the rRIC signals, the ridge width range was set to 10–50 s, the minimum ridge length to 3 s, and the signal-to-noise ratio threshold to 1. These parameters were selected empirically. An illustrative sensitivity analysis on a representative reference–sample pair showed that chromatographic landmark detection was largely preserved under moderate variations of the CWT parameters (Table S1).

For each peak detected in the rRIC, the drift-time profile recorded at the corresponding retention position was analyzed using the same CWT procedure and the Ricker wavelet as the mother wavelet. For these profiles, the ridge width range was set to 0.1–0.6 ms, the minimum ridge length to 0.14 ms, and the signal-to-noise ratio threshold to 1. When more than one drift-time peak was detected, only the most intense peak was retained. If no drift-time peak was detected, the corresponding chromatographic peak was excluded from further analysis for that sample. An analogous sensitivity analysis of the drift-time CWT parameters showed that peak selection remained unchanged under moderate variations in minimum ridge length and SNR, whereas the selected wavelet width range had a greater influence (Table S2).

The peak table of each sample was compared with that of the reference sample to establish the peak correspondence. Candidate matches were first identified based on an overlap in drift time. For each candidate, the correspondence was then assessed using the retention time of the peak apex. A tolerance of 20% was applied to the retention-time boundaries of the corresponding reference peaks. This tolerance was selected empirically. A sample peak was assigned to a reference peak only when both the drift time and the retention-time criteria were satisfied.

When a match was accepted, the peak table was updated with the identifier and the reference coordinates of the corresponding peak. Unmatched peaks were excluded from subsequent chromatographic correction. When several candidate peaks from the same sample could be assigned to a single reference peak, only the candidate with the smallest absolute difference in the retention time was retained.

Chromatographic peak detection and final landmark selection were treated as separate steps. A detected retention-time peak was retained as a spline landmark only when an unambiguous drift-time correspondence could also be established. Candidate peaks could fail to become landmarks because of an ambiguous drift-time assignment, insufficient chromatographic detectability, or the absence of a consistent counterpart in the reference chromatogram. Detection frequencies for the 15 reference peak classes are provided in Table S3.

The matched retention positions were then used as reference points for final chromatographic correction. For each sample, cubic spline interpolation was used to construct a continuous mapping between the retention positions of the matched peaks and the corresponding positions in the reference sample. The resulting transformation was applied to the entire retention-time axis. Because the mapping was defined by several matched peaks distributed across the chromatogram, it allowed residual changes in chromatographic displacement to be corrected locally while preserving the continuity of the transformed axis and its first and second derivatives. A total number of 9 matched peaks were obtained for every sample, allowing the spline correction to be applied in all cases. The monotonicity of the spline-based retention-time transformations was verified by evaluating their local slopes over the complete retention-time axis. The minimum local slope was positive for all 134 chromatograms, confirming that no local time axis inversions occurred (Table S4).

#### 2.2.3 Chromatographic Alignment Using Correlation Optimized Warping

Correlation optimized warping (COW) was used as a comparative chromatographic alignment method. COW divides the retention-time axis into segments and independently stretches or compresses each segment to maximize its correlation with the corresponding region of the reference chromatogram. The method is controlled by two main parameters: segment length, which determines the number of data points included in each segment, and slack, which limits the maximum change in the segment length during warping. These parameters determine the flexibility of the transformation and must therefore be selected for the dataset under study.^44^

COW was evaluated under three conditions: direct application to the preprocessed chromatograms, application after injection point correction, and application after injection point correction followed by parametric time warping. This design allowed for the influence of the initial correction stages on the subsequent COW alignment to be assessed.

COW was implemented in MATLAB R2024b using the version available in PLS_Toolbox 9.5 (Eigenvector Research, Inc., Manson, WA, USA). For each condition, segment length and slack were optimized using the *opt_cow* function, with search ranges of 20–200 data points for segment length and 2–50 data points for slack. The selected segment length and slack values were 66 and 23 for direct COW, 110 and 41 for injection point correction followed by COW, and 65 and 15 for injection point correction plus PTW followed by COW, respectively. The MATLAB code used for parameter optimization is provided in the Supporting Information.

#### 2.2.4 Quantitative Assessment of Chromatographic Alignment

Chromatographic alignment was evaluated from complementary perspectives by using both profile-based metrics and measures of peak correspondence and positional consistency.

Conventional alignment performance was evaluated using Pearson’s correlation, Euclidean similarity, simplicity, and peak factor. Pearson correlation and Euclidean similarity quantified global agreement between each aligned chromatogram and the reference.^45,46^ Simplicity assessed the structural consistency of the complete chromatographic dataset, whereas the peak factor quantified changes in overall signal magnitude introduced by the alignment transformation.^47^ The mathematical definitions of these metrics are provided in the Supporting Information.

Peak correspondence was evaluated using agreement and recovery metrics adapted from the precision and recall framework previously applied to chromatographic alignment against known positive peak pairs.^48^ For each recovered correspondence, the absolute retention-time deviation from the corresponding peak position identified by the proposed method in the same sample was also calculated. In addition, the residual dispersion of corresponding peak positions was quantified because variability in aligned retention times provides a direct measure of positional consistency after alignment.^49^ These complementary metrics were used because global chromatographic similarity, peak correspondence, and positional consistency represent distinct aspects of the alignment performance.

##### 2.2.4.1 Peak Correspondence Agreement and Recovery

A total of 15 reference peak classes were defined from peaks detected in the reference chromatogram after application of the proposed method. Peak detection was restricted to the interval from 70 to 920 s and used predefined thresholds for the relative prominence, minimum peak distance, and minimum peak width. The 15 peaks with the highest prominence were retained as the reference classes. These classes were used exclusively as an operational reference for comparing the correspondence patterns produced by different alignment workflows and were not assumed to represent chemically identified compounds or independent ground-truth correspondences. For each sample, peaks obtained with the proposed method were assigned to the reference classes using a monotonic sequence-matching procedure based on the retention time, normalized peak height, and normalized peak prominence.

Peaks detected after application of the other alignment workflows were subsequently matched to the correspondences identified by the proposed method in the same sample. This matching preserved the chromatographic peak order and was based on normalized peak height and normalized peak prominence. The temporal center of each reference class was defined as the median retention time of the corresponding peaks identified by the proposed method across the samples. Each matched peak from an evaluated workflow was then assigned to the reference class with the nearest temporal centers. Agreement with the operational reference was recorded when the assigned class coincided with the reference correspondence to which the peak was matched.

Agreement was calculated as the proportion of correctly assigned correspondences among those reference correspondences for which the evaluated workflow yielded a matched peak

2 $$Agreement=\frac{N_{correct}}{N_{\det ected}}$$

Recovery was calculated as the proportion of all available reference correspondences that were correctly recovered

3 $$Recovery=\frac{N_{correct}}{N_{reference}}$$

where *N*_correct_ is the number of matched peaks assigned to the correct reference class, *N*_detected_ is the number of reference correspondences for which the evaluated workflow yielded a matched peak, and *N*_reference_ is the total number of correspondences in the operational reference. Agreement is analogous to precision but is conditional on the correspondences detected by the evaluated workflow, whereas recovery is analogous to recall and quantifies the proportion of reference correspondences that were correctly preserved by each workflow. The operational reference comprised nine classes used as alignment anchors and six additional classes included only for evaluation for a total of 15 reference peak classes. Because the proposed method was used to establish the operational reference correspondences, it was excluded from the agreement and recovery comparisons.

##### 2.2.4.2 Paired Absolute Retention-Time Error

To complement the correspondence analysis, paired absolute retention-time error was used to quantify the positional deviation of each recovered correspondence. For each sample and peak class, the retention time obtained with the evaluated workflow was compared with that of the corresponding peak identified by the proposed method in the same sample. The error was calculated as

4 $$e_{\mathrm{ik}}=\left|t_{\mathrm{ik}}^{\left(w\right)}-t_{\mathrm{ik}}^{\left(\mathrm{ref}\right)}\right|$$

where *t*_*ik*_^(w)^ is the retention time of peak class *k* in sample *i* after application of the evaluated workflow, and *t*_*ik*_^(ref)^ is the retention time assigned to the same correspondence by the proposed method. Smaller values indicate greater positional agreement with the operational reference.

For each workflow, the distribution of paired absolute errors was summarized by using the median and the 90th percentile. The median represents the typical positional deviation, whereas the 90th percentile describes the magnitude of the larger deviations observed among the recovered correspondence. Because the proposed method defined the operational reference correspondences, it was not included in the paired absolute error comparisons.

##### 2.2.4.3 Within-Class Positional Dispersion

Residual within-class dispersion was quantified using a robust adaptation of the retention-time variability previously used to assess chromatographic alignment performance.^49^ For each workflow and peak class, dispersion was calculated using a robust estimate of the standard deviation

5 $$\sigma_{k,rob}=1.4826{median}_{i}\left(|t_{\mathrm{ik}}-{\mathrm{t̃}}_{k}|\right)$$

where *t*_*ik*_ is the retention time assigned to peak class *k* in sample *i*, and *t̃*_*k*_ is the median retention time of that class across samples. The scaling factor 1.4826 makes the median absolute deviation comparable to the standard deviation under a normal distribution.

For each workflow, only peak classes detected in at least 10 samples were included in the summary. Positional compactness was summarized as the median robust standard deviation across the included peak classes. Smaller values indicate a more compact distribution of the corresponding peak positions and, therefore, greater positional consistency after alignment. The proposed method was included in this assessment because the within-class dispersion quantifies the observed spread of aligned peak positions within each class.

## 3 Results

This section first presents the successive stages of the proposed alignment strategy and their qualitative effects on the GC-IMS data (Section 3.1). The proposed workflow is then compared with COW applied after different initial correction stages using both conventional profile-based metrics and the peak correspondence measures described in Section 2.2.4 (Section 3.2).

### 3.1 Application of the Alignment Strategy

A total of 134 GC-IMS measurements of the pooled urine sample were included in the alignment analysis. Sample 58 was selected as the reference using the cross-correlation procedure described in Section 2.2.1. Figure 3 shows its reversed reactant-ion peak chromatogram. The profile was dominated by intense, closely spaced peaks at shorter retention times, whereas peaks at longer retention times were generally smaller and more widely separated. The injection point of the reference chromatogram was located at 167.31 s. Each sample was shifted so that its injection point coincided with this position. Because the required shifts differed among samples, the retention-time range was restricted to the interval shared by all chromatograms, resulting in a final upper limit of 1234.35 s.

**Figure 3:**
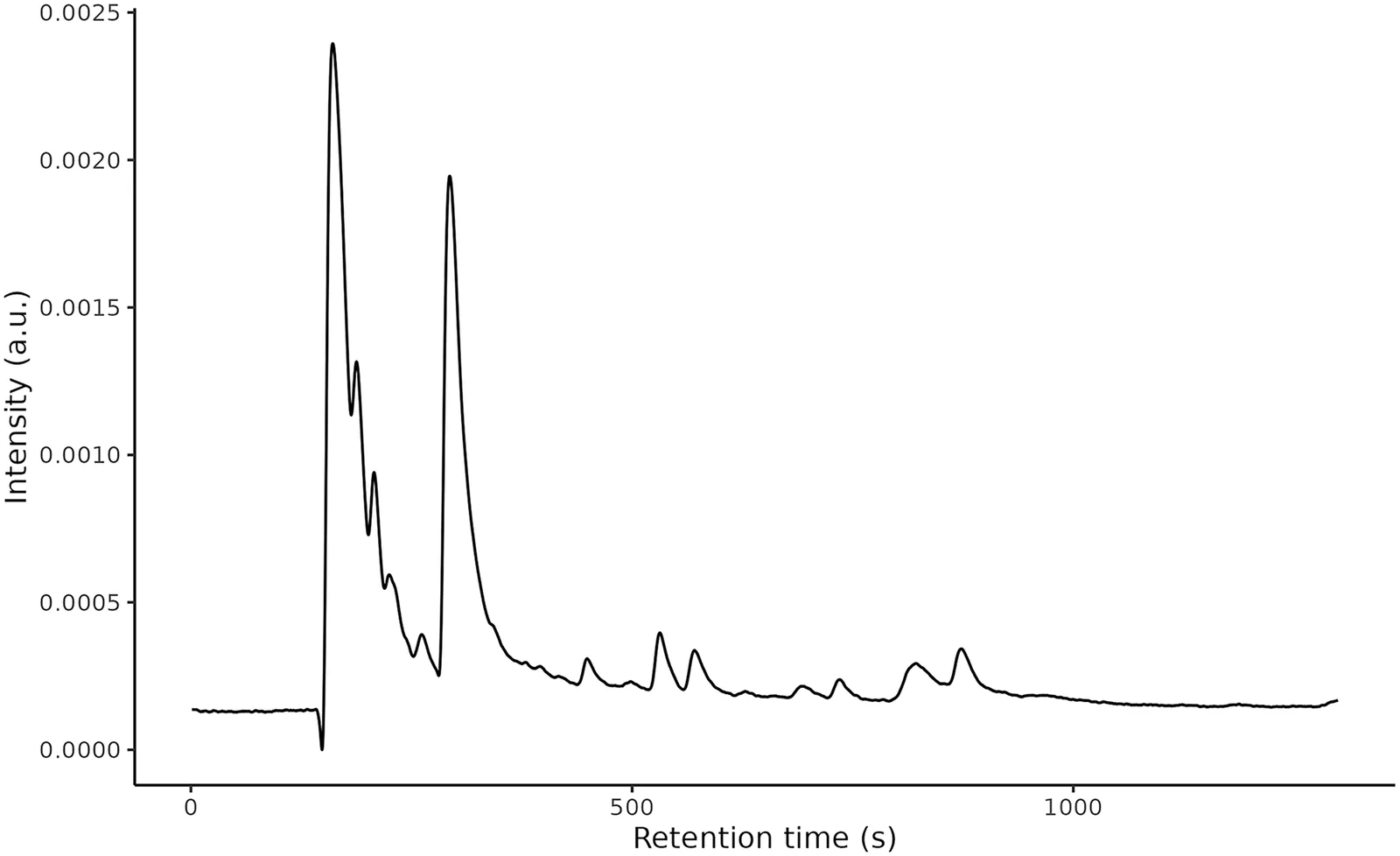
Reversed reactant-ion peak chromatogram of the reference sample (sample 58), showing a higher density of intense peaks at shorter retention times.

After injection point correction, the retention-time axis was further adjusted by using parametric time warping with a second-order polynomial. This rigid transformation was intended to correct the dominant global deformation while preserving the monotonicity of the retention-time axis. The resulting warping functions were monotonic for all of the samples. The correction improved the alignment of the most intense peaks at shorter retention times, although residual misalignment remained at longer retention times. The drift-time axis was then corrected by aligning the reactant-ion peak of each sample with its position in the total ion spectrum of the reference sample at 7.55 ms. A multiplicative correction was applied to the drift-time axis. The correction factor had a mean of 1.00 and a standard deviation of 4.2 × 10^–3^, indicating only minor variation in the drift-time dimension across samples.

Peaks were detected along both the retention-time and drift-time dimensions using the Continuous Wavelet Transform. Corresponding peaks were then identified across samples using information from both dimensions. Nine peak correspondences were identified, and their positions in the reference GC-IMS map are shown in Figure 4. Six of these correspondences were located at longer retention times, where shift correction and polynomial prealignment had not provided satisfactory alignment. The nine correspondences were therefore used as anchors for the final refinement of the retention-time axis by cubic spline interpolation.

**Figure 4:**
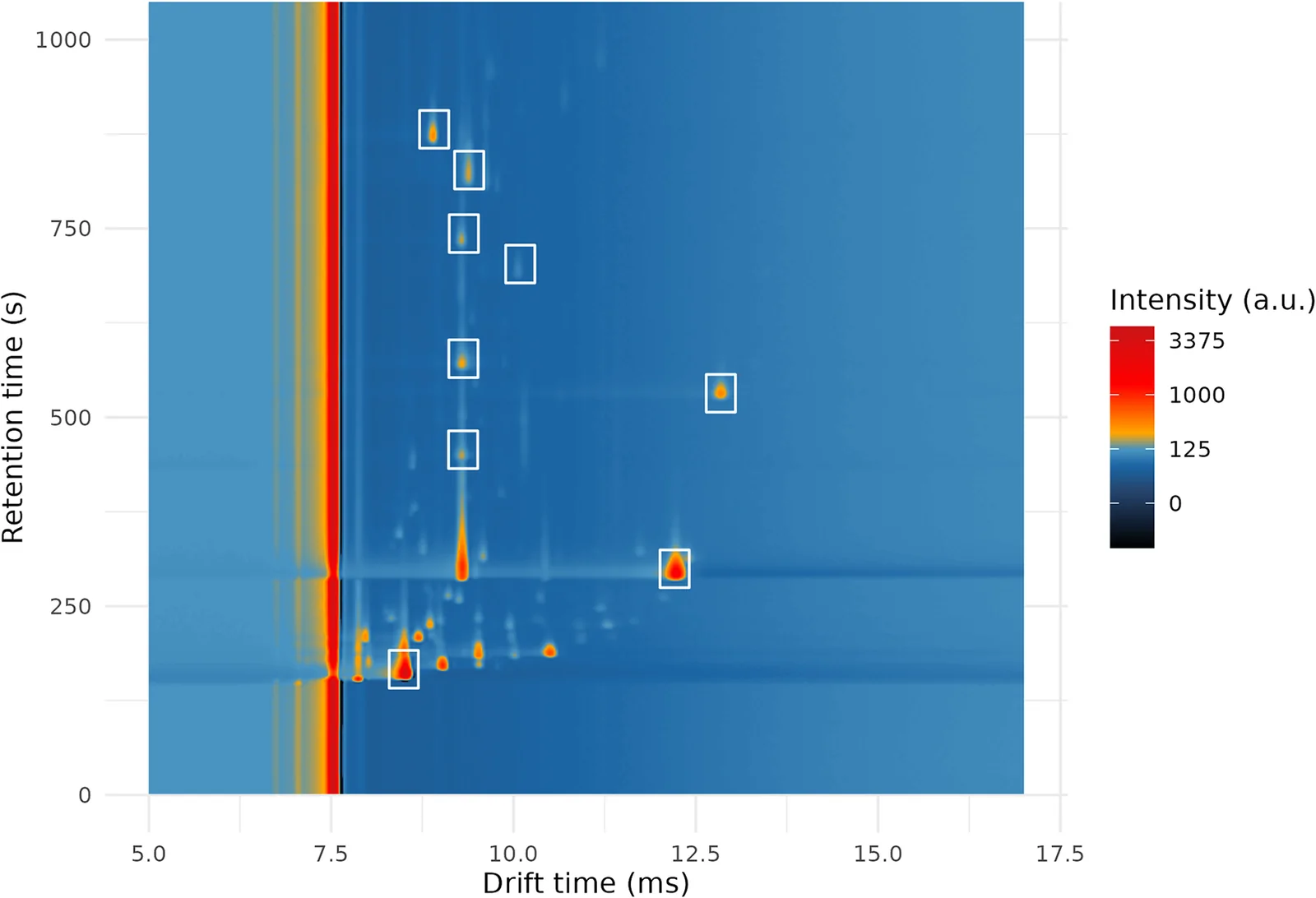
GC-IMS map of the reference sample. White rectangles indicate the nine peak correspondences used as anchors for the final refinement of the retention-time axis by cubic spline interpolation.

Figure 5 shows the progressive effect of the different retention-time correction stages on the reversed reactant-ion peak chromatograms. Before correction, the chromatograms displayed substantial displacement across the full retention-time range, with increasing divergence at longer retention times (Figure 5a). Injection point correction aligned the initial region of the chromatograms but did not remove the progressive deformation observed later in the run (Figure 5b). PTW further improved the overall agreement and made the common chromatographic structure more apparent, although residual misalignment remained beyond approximately 500 s (Figure 5c). After the final spline refinement, the chromatograms showed close agreement across the complete retention-time range, with the most evident improvement occurring between approximately 500 and 900 s (Figure 5d).

**Figure 5:**
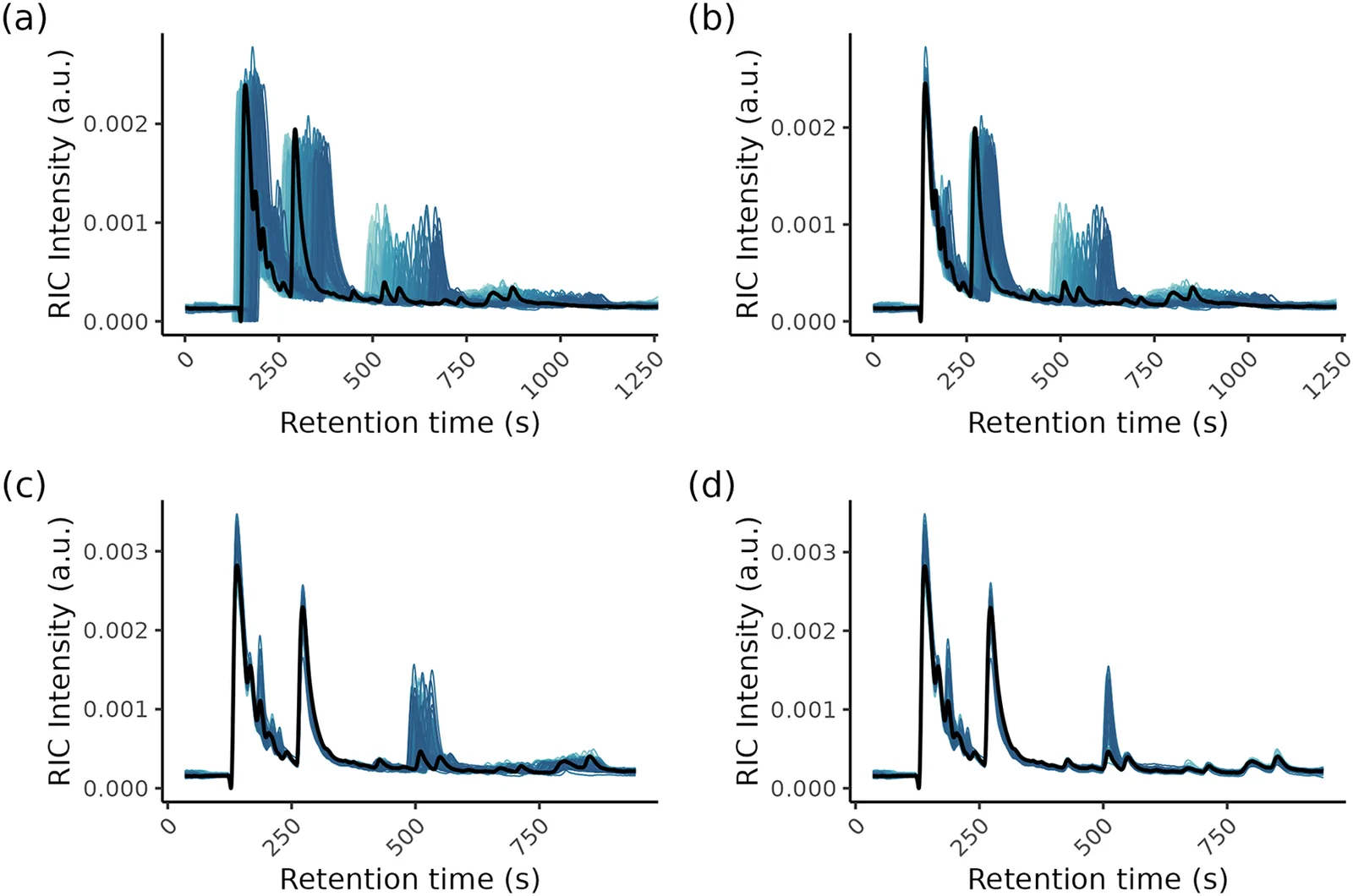
Reversed reactant-ion peak chromatograms of the samples (blue) and the reference sample (black) at successive stages of the proposed workflow: (a) before alignment, (b) after injection point correction, (c) after injection point correction and PTW prealignment, and (d) after final spline refinement guided by peak correspondences supported by the drift-time dimension.

The combined effect of the retention-time and drift-time corrections is visible in the averaged GC-IMS maps shown in Figure 6. Before alignment, positional variability caused the two-dimensional peak regions to appear broadened and diffuse (Figure 6a). Injection point correction mainly sharpened peaks at shorter retention times (Figure 6b). After PTW prealignment and drift-time correction, the peak regions became more compact in both dimensions, although residual dispersion remained at longer retention times (Figure 6c). Following the final spline refinement, peaks across the complete retention-time range appeared sharper and more clearly defined, including low-intensity regions that had remained diffuse after prealignment (Figure 6d).

**Figure 6:**
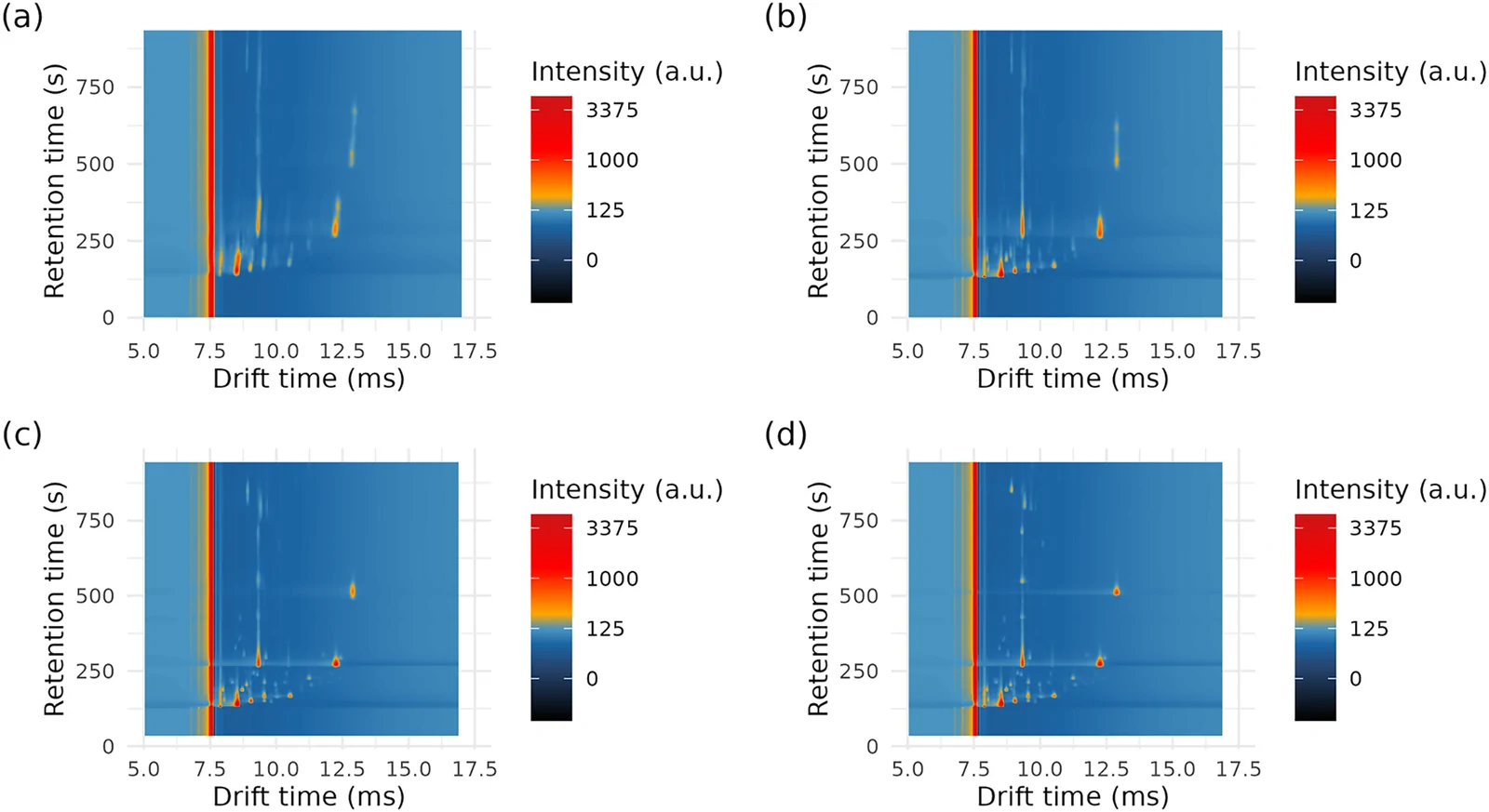
Averaged GC-IMS maps at successive stages of the proposed workflow: (a) before alignment, (b) after injection point correction, (c) after injection point correction, PTW prealignment, and drift-time correction, and (d) after final spline refinement. Peak regions become progressively sharper and more clearly defined across the alignment stages.

### 3.2 Assessment of the Alignment

Figure 7 compares the alignments obtained with COW under three initial correction conditions. The original chromatograms are shown for reference in Figure 7a. When COW was applied directly to the unaligned chromatograms, residual displacement remained in several regions, particularly between approximately 500 and 750 s (Figure 7b). Correcting the injection point before applying COW improved the alignment, but in approximately half of the samples, the penultimate peak was incorrectly matched to the final peak (Figure 7c). Applying COW after shift correction and PTW did not resolve this correspondence error and increased peak dispersion at longer retention times (Figure 7d). The warping functions obtained with the COW were monotonic in all three configurations.

**Figure 7:**
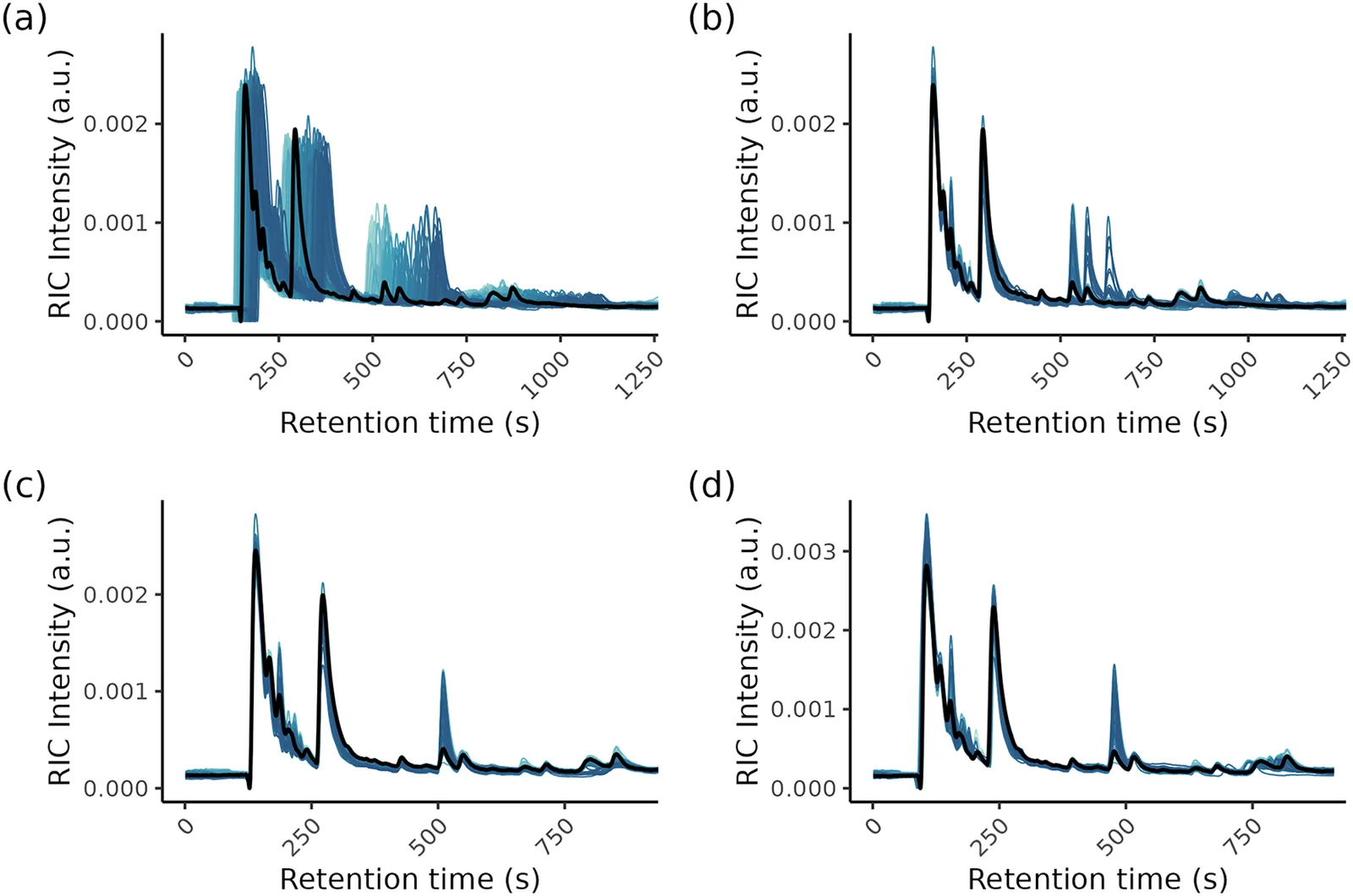
Reversed reactant-ion peak chromatograms of the samples (blue) and the reference sample (black) before and after COW under different initial correction conditions: (a) before alignment, (b) after direct application of COW to the unaligned chromatograms, (c) after injection point correction followed by COW, and (d) after injection point correction and PTW prealignment followed by COW.

The conventional alignment metrics are summarized in Table 1. Correlation, Euclidean similarity, and simplicity increased progressively across the stages of the proposed workflow. The largest improvement occurred after injection point correction, followed by a further increase after PTW prealignment and a smaller improvement after the final spline refinement. The peak factor remained close to 1 after shift correction but decreased after PTW, indicating a larger change in overall signal magnitude during the nonlinear prealignment stage. The final refinement produced only a minimal additional change in the metric.

**Table 1:** Conventional Alignment Metrics for the Unaligned Data, the Successive Stages of the Proposed Workflow, and the Three Cow Configurations^a^

method	correlation	similarity	simplicity	peak factor
no alignment	0.613 ± 0.231	0.359 ± 0.234	0.5466	1.0000
shift correction	0.870 ± 0.014	0.623 ± 0.158	0.8297	0.9995
prealignment (shift correction + PTW)	0.977 ± 0.013	0.809 ± 0.054	0.9664	0.9687
proposed workflow	0.983 ± 0.012	0.834 ± 0.056	0.9823	0.9686
COW	0.980 ± 0.014	0.838 ± 0.053	0.9744	0.9973
shift correction + COW	0.983 ± 0.011	0.844 ± 0.047	0.9797	0.9975
shift correction + PTW + COW	0.981 ± 0.015	0.827 ± 0.061	0.9788	0.9678

a Pearson correlation and Euclidean similarity were calculated individually for each sample and are reported as mean ± standard deviation across the dataset. Simplicity and peak factor were calculated for the complete dataset.

The three COW configurations also produced high correlation, Euclidean similarity, and simplicity values. Shift correction followed by COW achieved the highest mean correlation and Euclidean similarity, whereas COW applied after shift correction and PTW produced slightly lower values and a peak factor comparable to that obtained after PTW prealignment. Overall, the conventional metrics indicated a high degree of similarity for several workflows but did not distinguish the correspondence errors observed in Figure 7.

Figure 8 presents the results obtained with correspondence-based metrics. In the unaligned data, agreement and recovery were 48.2% and 46.8%, respectively, and the median paired positional deviation was 24.18 s (Figure 8a,b). Injection point correction increased the agreement to 67.8% and recovery to 66.5% while reducing the median deviation to 12.09 s. After shift correction and PTW, agreement and recovery reached 84.5% and 84.2%, respectively, and the median deviation decreased to 2.34 s. These results indicate a progressive improvement in peak correspondence across the prealignment stages.

**Figure 8:**
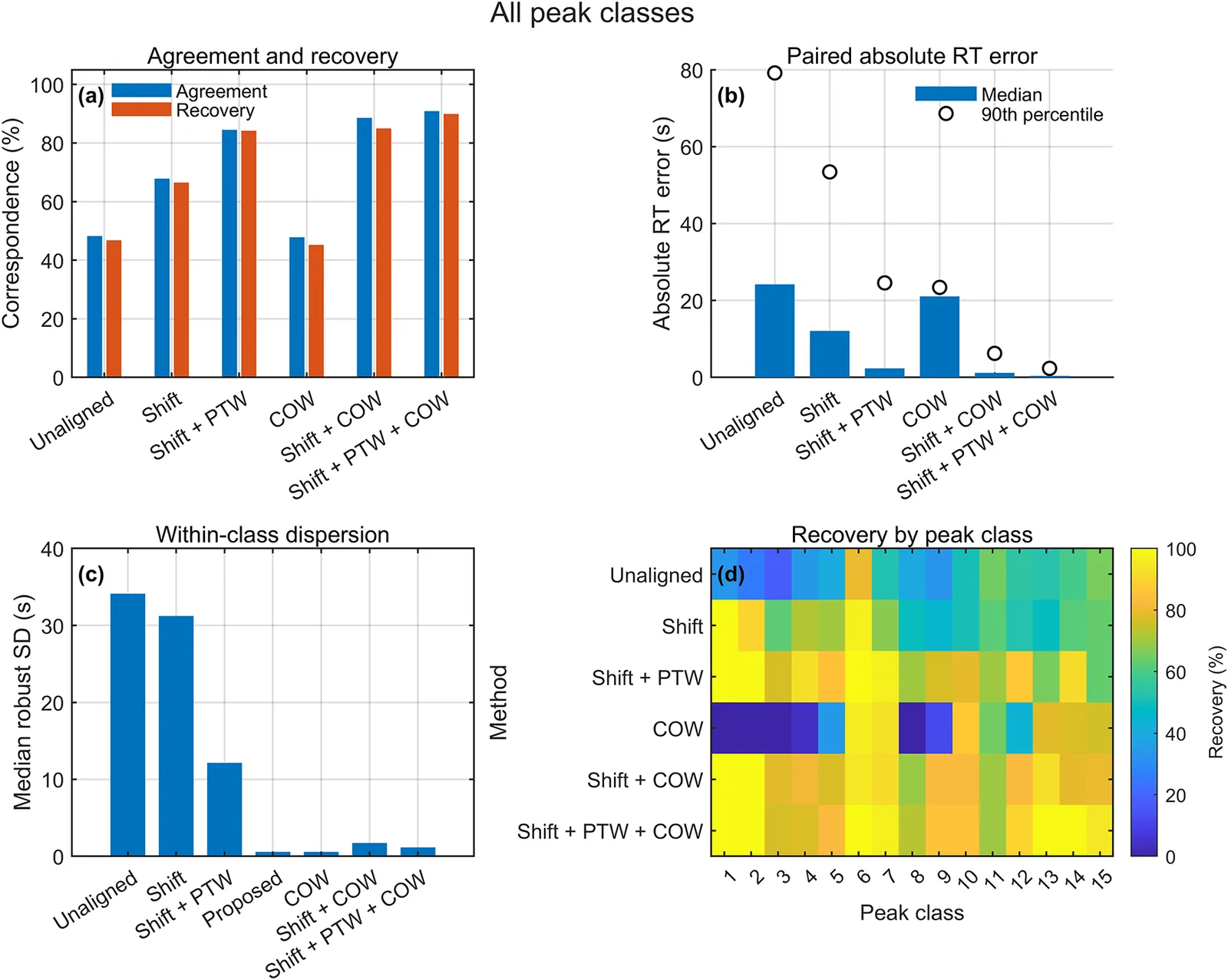
Assessment of peak correspondence and positional consistency for the different alignment workflows. (a) Agreement and recovery relative to the operational reference correspondences. (b) Paired absolute retention-time error for recovered correspondences, summarized by the median and the 90th percentile. (c) Within-class positional dispersion, expressed as the median robust standard deviation across the included peak classes. (d) Recovery for each of the 15 reference peak classes. The operational reference comprised nine classes used as alignment anchors and six additional classes used only for evaluation.

Direct application of COW yielded agreement and recovery values of 47.8% and 45.2%, respectively, which were similar to those obtained for unaligned data. The median paired deviation was 21.06 s. Correcting the injection point before COW increased the agreement to 88.6% and recovery to 85.0% while reducing the median deviation to 1.17 s. When COW was applied after shift correction and PTW, agreement reached 90.9%, recovery reached 89.9%, and the median deviation decreased to 0.39 s.

Within-class dispersion followed a different pattern (Figure 8c). The median robust standard deviation decreased from 34.1 s in the unaligned data to 31.2 s after shift correction and 12.1 s after shift correction and the PTW. After the final spline refinement, the time decreased further to 0.58 s. Direct application of COW produced the same dispersion value of 0.58 s despite its low agreement and recovery. Thus, COW generated compact peak distributions, even when the resulting clusters did not preserve the operational reference correspondences.

The recovery heat map shows that performance also varied among peak classes (Figure 8d). Direct application of COW yielded low recovery for several classes, whereas recovery generally increased when COW was preceded by shift correction or by the complete prealignment procedure. These results indicate that high global profile similarity and low positional dispersion do not necessarily imply the preservation of consistent peak correspondences.

## 4 Discussion

The results support a sequential strategy for aligning GC-IMS data. The different stages address complementary components of retention-time variability. Injection point correction removes the dominant initial displacement, PTW corrects the main global deformation, and the final spline refinement uses peak correspondences supported by both the retention-time and drift-time dimensions to correct the remaining local deviations. Each stage therefore addresses residual misalignment left by the preceding correction.

The comparison with COW further illustrates the importance of the initial correction before flexible warping. When COW was applied directly to the unaligned chromatograms, it produced high values for the conventional profile-based metrics and low within-class dispersion, but the agreement and recovery remained similar to those of the unaligned data. Correcting the injection point before COW markedly improved peak correspondence, and the best COW results were obtained when both shift correction and PTW were applied beforehand. These results suggest that reducing the dominant global deformation before applying a flexible warping method helps to avoid compact peak groupings that do not preserve the operational reference correspondences.

The comparison also highlights an important distinction between the global profile similarity and peak correspondence. Correlation, Euclidean similarity, and simplicity were high for several workflows and differed only slightly among the final alignment strategies. However, these metrics did not capture the correspondence errors observed in Figure 7 or the low agreement and recovery obtained when COW was applied directly to the unaligned chromatograms. Likewise, low within-class dispersion did not necessarily indicate that operational reference correspondences had been preserved. Alignment quality should therefore not be evaluated exclusively in terms of profile similarity or peak compactness but should also consider whether corresponding chromatographic features remain correctly associated across samples.

An important feature of the proposed method is the use of spectral information from the drift-time dimension to support the identification of corresponding chromatographic peaks. Retention time alone may be insufficient when peaks are closely spaced, partially overlap, or are affected by local distortions. In these situations, the mobility dimension provides complementary information that helps to distinguish peaks with similar retention times. This was particularly useful at longer retention times, where residual misalignment remained after shift correction and PTW and where several of the selected anchor correspondences were located. The final spline refinement therefore relies on peak correspondences supported by both dimensions rather than on the chromatographic shape alone.

The use of spectral information also reduces the need to introduce internal standards solely for alignment purposes. Avoiding such standards simplifies sample preparation and prevents intentional modification of sample composition. This may be particularly relevant in GC-IMS, where ionization and detection are influenced by competition for reactant ions. When two compounds elute simultaneously, the compound with the higher proton affinity may suppress the response of the other. Consequently, an added standard may interfere with an endogenous compound, or its own signal may be suppressed, reducing its reliability as an alignment reference. Using naturally occurring peak correspondences supported by both retention time and drift time avoids this potential source of interference.

Several limitations should be considered when interpreting these results. First, the correspondence analysis used peak classes defined from the proposed method as an operational reference. These classes were not chemically identified and do not constitute independent ground truth for peak correspondence. Agreement, recovery, and paired positional deviation could be calculated for the other workflows, but these metrics do not provide an independent validation of the proposed alignment itself. The inclusion of six additional classes used only for evaluation extends the assessment beyond the peaks used for spline refinement, but does not remove this limitation. Validation using mixtures containing compounds of known identity would provide a stronger independent assessment of correspondence and should be considered in future studies. Second, the study was performed on repeated measurements of a single pooled urine sample. This controlled design was useful for assessing instrumental retention-time variability because the underlying sample composition was expected to remain approximately constant across measurements. However, it does not establish the performance of the method when substantial biological or compositional differences are present among samples. Further evaluation is therefore required using heterogeneous biological samples and GC-IMS datasets acquired under different experimental conditions. Finally, the proposed method depends on the detection of sufficiently reliable peak correspondences in both dimensions. Its performance may decrease in datasets with sparse signals, severe peak overlap, or limited mobility resolution.

Despite these limitations, the study demonstrates the value of combining chromatographic prealignment with peak correspondences supported by the mobility dimension. The proposed strategy progressively corrects global and local retention-time variability while reducing the risk of alignment of unrelated peaks. More generally, the results emphasize that chromatographic alignment should preserve peak correspondence rather than optimize the profile similarity or positional compactness alone.

## 5 Conclusions

This study presents a chromatographic alignment strategy for GC-IMS data that combines injection point correction, parametric time warping, and a final spline refinement guided by spectral information from the drift-time dimension. The successive correction stages progressively improved alignment across the retention-time range, with the final refinement providing the most evident improvement at longer retention times.

The comparison with COW showed that the initial correction stages strongly influenced the preservation of the peak correspondences. Direct application of COW produced high global similarity and low positional dispersion, although agreement and recovery remained similar to those of the unaligned data. Its performance improved markedly when the shift correction and PTW were applied beforehand. These results show that favorable values of global similarity or peak compactness do not necessarily imply the preservation of the operational reference correspondences. Chromatographic alignment should therefore be assessed by using both profile-based metrics and measures of peak correspondence.

By using information from both the retention-time and drift-time dimensions, the proposed workflow supports the identification of suitable peak correspondences without introducing internal standards solely for alignment. The method therefore provides a practical strategy for correcting global and local retention-time variability in repeated GC-IMS measurements. Further evaluation using samples with greater compositional diversity and data acquired under different experimental conditions will be required to establish its broader applicability.

## Supplementary Material



## Notes

Mathematical definitions of the conventional chromatographic alignment metrics; sensitivity analysis of CWT peak-detection parameters; landmark availability, selection, and ablation analysis; spline monotonicity assessment; and MATLAB code for COW parameter optimization (PDF)
