# Supplementary material for: Improved Chromatographic Alignment of GC-IMS Data Using Spectral Information
Source: ACS Meas Sci Au. 2026 Oct 2;6(5):1916–28. doi: 10.1021/acsmeasuresciau.6c00271 (PMC13693273; doi:10.1021/acsmeasuresciau.6c00271)
Supplement: Supplementary file 1 [file tg6c00271_si_001.pdf]

Improved Chromatographic Alignment of GC-IMS Data Using Spectral Information

Luis Fernández[1,2], Gema Guedes de la Cruz[2], Eduardo Caballero[2], Eva Martín-López[2], Albert Vidal[2], Javier Alonso-Valdesueiro[1,2], Josep Gumà[3], Raquel Cumeras[4], Antonio Pardo[1,*], Santiago Marco[1,2]

[1] Department of Electronics and Biomedical Engineering, Universitat de Barcelona, 08028 Barcelona, Spain

[2] Institute for Bioengineering of Catalonia (IBEC), The Barcelona Institute of Science and Technology, 08028 Barcelona, Spain

[3] Institut d'Oncologia de la Catalunya Sud (IOCS), Hospital Universitari Sant Joan de Reus, Facultat de Medicina i Ciències de la

Salut, Universitat Rovira i Virgili, 43204 Reus, Spain.

[4] Institut de Recerca Biomèdica Catalunya Sud (IRBCatSud), 43204 Reus, Spain.

*Corresponding author: a.pardo@ub.edu

Supporting information

Index

S1. Conventional chromatographic alignment metrics	Pag 3
S2. Sensitivity analysis of CWT peak-detection parameters	Pag 5
S3. Landmark availability, selection, and ablation analysis	Pag 7
S4. Spline monotonicity assessment	Pag 11
S5. MATLAB code for COW optimization	Pag 12

S1. Conventional chromatographic alignment metrics

Euclidean distance and similarity

Euclidean distance was used to quantify the overall difference between each aligned chromatogram and the reference. Let **y** = ($y_1,\ldots,y_m$) denote the reference chromatogram and $\mathbf{x}^{(w)}$ = ($x_1^{(w)},\ldots, x_m^{(w)}$) the corresponding chromatogram after alignment. The Euclidean distance was calculated as:

$$d = \sqrt{\sum_{j=1}^{m}\left(y_j - x_j^{(w)}\right)^2} \tag{SE1}$$

Smaller values of d indicate greater similarity between the aligned chromatogram and the reference. Following the general transformation of an unbounded distance into a normalized similarity measure, Euclidean distance was converted to similarity as:

$$S_E = 1 - \frac{d}{d_{max}} \tag{SE2}$$

In the present study, $d_{\max}$ was defined as the largest Euclidean distance observed between the original unaligned chromatograms and the reference. Similarity values closer to 1 indicate greater agreement with the reference, whereas lower values indicate greater overall disagreement.

Pearson correlation coefficient

The Pearson correlation coefficient was used to quantify the linear similarity between each aligned chromatogram and the reference chromatogram. This coefficient is commonly used both as an objective function and as a measure of overall alignment quality in chromatographic signal alignment. It was calculated as:

$$r = \frac{\sum_{j=1}^{m}\left(x_j^{(w)} - \bar{x}^{(w)}\right)\left(y_j - \bar{y}\right)}{\sqrt{\sum_{j=1}^{m}\left(x_j^{(w)} - \bar{x}^{(w)}\right)^2}\sqrt{\sum_{j=1}^{m}\left(y_j - \bar{y}\right)^2}} \tag{SE3}$$

where $\bar{x}^{(w)}$ and $\bar{y}$ denote the mean intensities of the aligned and reference chromatograms, respectively. The coefficient ranges from -1 to 1, with values closer to 1 indicating greater linear similarity between the two profiles.

Simplicity value

The simplicity value was used to assess the structural consistency of the complete set of chromatograms after alignment. It is based on the singular value decomposition of the uncentered data matrix $\mathbf{X} \in \mathbb{R}^{n\times m}$, in which rows represent samples and columns represent retention time points. It was calculated as:

$$Simplicity = \frac{\sum_{r=1}^{R} \sigma_r^4}{\left(\sum_{i=1}^{n} \sum_{j=1}^{m} x_{ij}^2\right)^2} \tag{SE4}$$

where σ_r denotes the r-th singular value of $\mathbf{X}$, x_{ij} is the intensity at retention time point j in sample i, and R is the number of singular values retained in the calculation. Because all samples were repeated measurements of the same pooled material, a single dominant chromatographic pattern was expected, and only the first singular value was retained (R = 1). Higher simplicity values indicate greater dominance of this common pattern across the dataset.

Peak factor

The peak factor was used to quantify changes in overall signal magnitude introduced by chromatographic alignment. This metric has been proposed as an indirect measure of peak preservation because changes in the chromatographic profile affect its Euclidean norm. For each sample, the Euclidean norms of the chromatogram before and after alignment were compared. The peak factor was calculated as:

$$Peak\ factor = \frac{1}{n}\sum_{i=1}^{n}\left[1 - \min\left(c_i, 1\right)^2\right], \qquad c_i = \left|\frac{\|x_i^{(w)}\| - \|x_i\|}{\|x_i\|}\right| \tag{SE5}$$

where $\boldsymbol{x}_i$ and $\boldsymbol{x}_i^{(w)}$ denote the chromatogram of sample i before and after alignment, respectively, and $\|\cdot\|$ denotes the Euclidean norm. The metric ranges from 0 to 1, with values closer to 1 indicating smaller changes in overall signal magnitude after alignment, whereas lower values indicate larger changes.

S2. Sensitivity analysis of CWT peak-detection parameters

An illustrative sensitivity analysis was performed to examine the influence of the empirically selected Continuous Wavelet Transform (CWT) parameters used for chromatographic and drift-time peak detection. The analysis used the reference measurement and one representative sample for which the nine spline-landmark correspondences had been verified.

For chromatographic peak detection in the reversed reactant ion peak chromatogram (rRIC), the nominal CWT settings were a wavelet-width range of 10–50 s, a minimum ridge length of 3 s, and a signal-to-noise ratio (SNR) threshold of 1. Sensitivity was evaluated by varying one parameter at a time while keeping the remaining settings fixed. The width range was varied by ±20% (8–40 s and 12–60 s), the minimum ridge length between 2 and 4 s, and the SNR threshold between 0.75 and 1.25. With the nominal settings, all nine validated chromatographic landmarks were detected in both the reference measurement and the representative sample. Moderate changes in the CWT parameters had only a limited effect on landmark detection. Eight of the nine validated landmark pairs were recovered after either a 20% decrease or increase in the wavelet-width range, after changing the minimum ridge length to 2 or 4 s, and when the SNR threshold was increased to 1.25. All nine landmark pairs were recovered when the SNR threshold was reduced to 0.75 (Table S1).

***Table S1**. Recovery of the nine validated chromatographic landmark correspondences under moderate variation of the CWT parameters in a representative reference–sample pair.*

Configuration	Reference landmarks detected	Sample landmarks detected	Landmark pairs detected in both
Nominal	9/9	9/9	9/9
Width −20%	9/9	8/9	8/9
Width +20%	9/9	8/9	8/9
Ridge length 2 s	9/9	8/9	8/9
Ridge length 4 s	9/9	8/9	8/9
SNR 0.75	9/9	9/9	9/9
SNR 1.25	9/9	8/9	8/9

An analogous analysis was performed on the drift-time profiles extracted at the validated chromatographic landmark positions. The nominal CWT settings were a wavelet-width range of 0.10–0.60 ms, a minimum ridge length of 0.14 ms, and an SNR threshold of 1. The width range was varied by ±20% (0.08–0.48 ms and 0.12–0.72 ms), the minimum ridge length between 0.10 and 0.18 ms, and the SNR threshold between 0.75 and 1.25. With the nominal settings, the same drift time peak was selected for all nine validated landmark correspondences in both measurements. The selected peak also remained unchanged for all nine correspondences when either the minimum ridge length or the SNR threshold was varied. Variation of the wavelet width range had a greater influence. After either a 20% decrease or a 20% increase in the nominal width range, the same drift time peak was retained for eight of the nine landmarks in the reference measurement and seven of the nine landmarks in the representative sample. Six of the nine correspondences remained unchanged in both measurements (Table S2).

These results showed limited sensitivity to moderate changes in the tested CWT parameters for this representative measurement pair. Chromatographic landmark detection was largely preserved across the tested configurations, whereas drift time peak selection showed a greater dependence on the selected wavelet-width range. Because the analysis was performed on a single representative pair of measurements, its interpretation is limited to this illustrative case.

Table S2. *Stability of drift-time peak selection for the nine validated landmark correspondences under moderate variation of the CWT parameters in a representative reference–sample pair.*

Configuration	Reference DT peaks unchanged	Sample DT peaks unchanged	Correspondences unchanged in both
Nominal	9/9	9/9	9/9
Width −20%	8/9	7/9	6/9
Width +20%	8/9	7/9	6/9
Ridge length 0.10 ms	9/9	9/9	9/9
Ridge length 0.18 ms	9/9	9/9	9/9
SNR 0.75	9/9	9/9	9/9
SNR 1.25	9/9	9/9	9/9

S3. Landmark availability, selection, and ablation analysis

Detection frequency

The alignment workflow distinguishes between chromatographic peak detection and final landmark selection. Peaks were first detected in the retention time profile, but only those for which an unambiguous correspondence could also be established in the drift-time dimension were retained as spline landmarks.

To characterize peak availability across the dataset, the 15 reference chromatographic peak classes used in the correspondence assessment were examined individually. Detection frequency varied among classes (Table S3). All nine spline landmarks used for chromatographic alignment were detected in all 134 chromatograms, whereas the remaining reference peak classes were less persistent. Peak class 11 showed the lowest detection frequency and was observed in 66/134 chromatograms, while peak class 3 was detected in 121/134 chromatograms.

The main causes of landmark rejection occurred at different stages of the workflow. In the early part of the chromatogram, approximately 140–270 s on the reference time axis, several chromatographic features are located within a dense retention- and drift-time region. In this region, some peaks could be detected chromatographically but were excluded when a unique drift time correspondence could not be assigned. In the intermediate region, particularly around 480 and 610 s on the reference time axis, variation in peak intensity caused some features to fall below the chromatographic detection criterion in individual measurements. At longer retention times, additional peaks were occasionally present beyond approximately 850 s, but these were not consistently observed across samples and therefore lacked a corresponding reference feature. Ambiguous or inconsistent candidates were excluded rather than forced into the landmark set.

Table S3. *Detection frequency of the 15 reference chromatographic peak classes*

**Peak class**	**Approx. RT in reference (s)**	**Final spline landmark**	**Detected chromatograms**	**Detection frequency (%)**
**1**	139.62	**Yes**	134	100.0
2	166.53	No	131	97.8
3	186.81	No	121	90.3
4	210.21	No	111	82.8
5	240.24	No	114	85.1
**6**	272.22	**Yes**	134	100.0
**7**	428.22	**Yes**	134	100.0
8	476.78	No	114	85.1
**9**	510.51	**Yes**	134	100.0
**10**	550.68	**Yes**	134	100.0
11	608.40	No	66	49.3
**12**	671.97	**Yes**	134	100.0
**13**	714.09	**Yes**	134	100.0
**14**	800.67	**Yes**	134	100.0
**15**	852.15	**Yes**	134	100.0

Landmark-ablation sensitivity analysis

To examine the effect of reduced landmark coverage, an illustrative ablation analysis was performed on one representative chromatogram for which all nine spline-landmark correspondences had been verified. All possible subsets retaining 4, 5, 6, 7, 8, or all 9 landmarks were evaluated. This resulted in 126, 126, 84, 36, 9, and 1 configurations, respectively.

For each configuration, the chromatogram was realigned using cubic spline interpolation. All resulting transformations were monotonic. Alignment performance was evaluated using the median moving-window shape NRMSE and the positional error of reference peak classes not used as spline landmarks. The latter provides a measure of how landmark removal affects chromatographic features that do not directly constrain the spline.

Alignment quality degraded progressively as the number of retained landmarks decreased. The median moving-window shape NRMSE increased from 0.227 with nine landmarks to 0.368 with four landmarks (Figure S1). Similarly, the median positional error of non-landmark peak classes increased from 0 s with nine landmarks to 1.56 s with four landmarks (Figure S2). The dispersion of both metrics also increased for configurations containing fewer landmarks, indicating a greater dependence on the spatial distribution of the remaining landmarks when landmark coverage was reduced.

These results indicate that landmark density and spatial coverage affect the stability of the spline correction, even when the resulting warping remains monotonic.

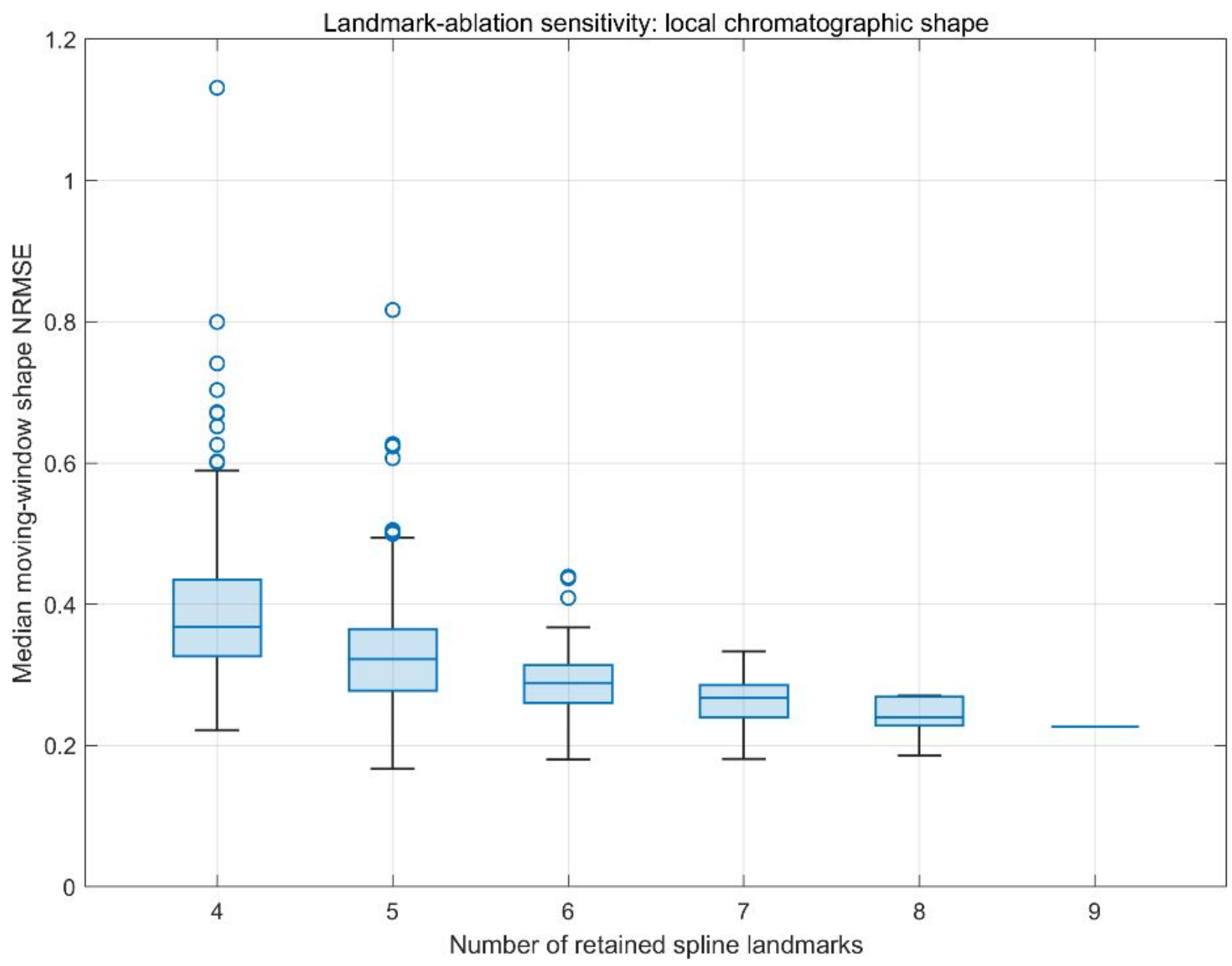

Figure S1. *Sensitivity of local chromatographic shape to spline-landmark removal. The median moving-window shape NRMSE increased as the number of retained landmarks decreased. Each distribution represents all possible landmark subsets of the indicated size.*

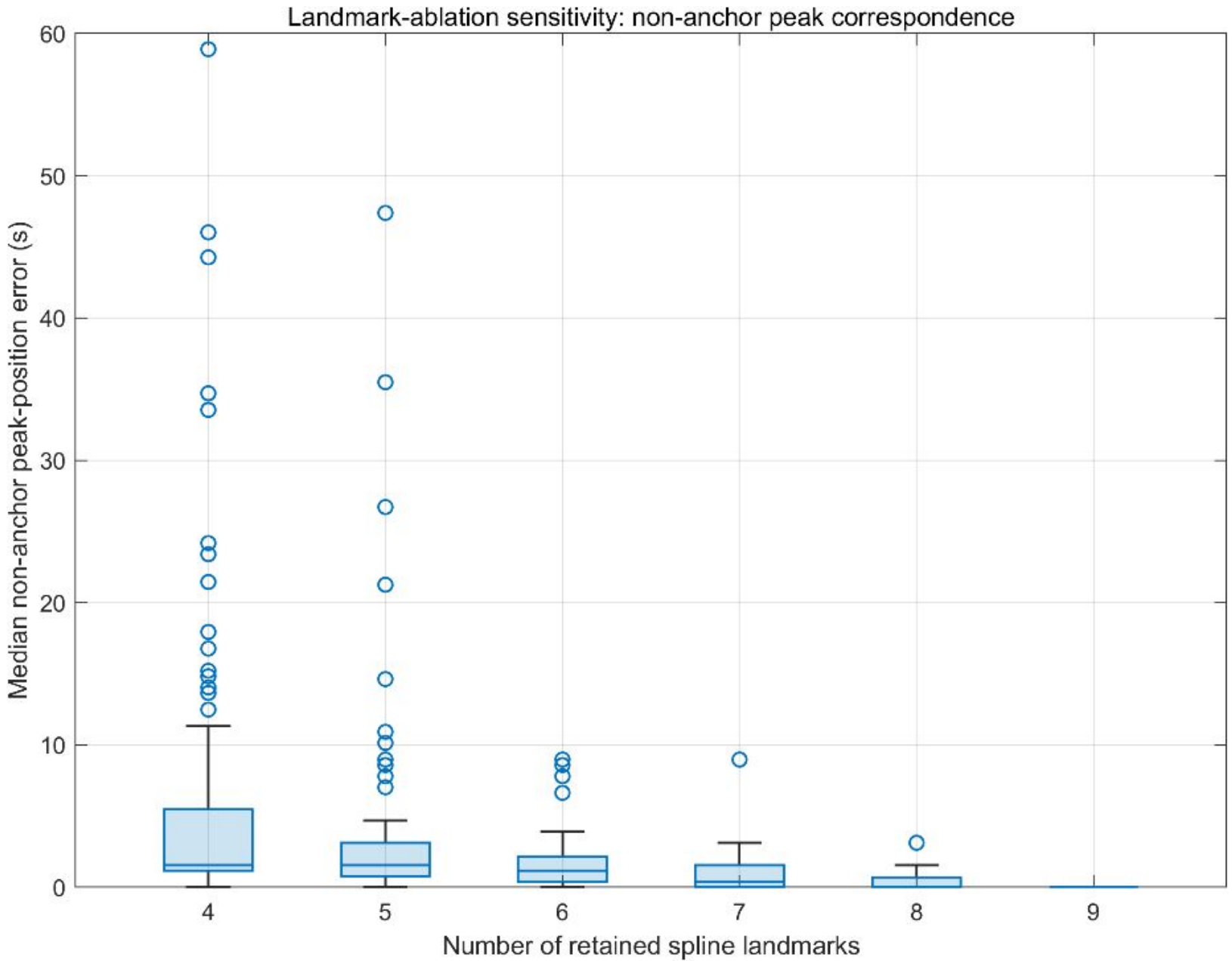

Figure S2. *Sensitivity of non-landmark peak correspondence to progressive spline-landmark removal. All possible subsets retaining 4–9 of the nine validated landmarks were evaluated for a representative chromatogram. The ordinate shows the median positional error of peak classes not used as spline landmarks, relative to the alignment obtained using the complete nine-landmark set. Error and variability increased as landmark coverage decreased.*

S4. Spline monotonicity assessment

The monotonicity of the spline-based retention-time transformations was assessed by evaluating the local slope of each transformation over the complete retention time axis. For each chromatogram, the minimum local slope was recorded as a conservative indicator of monotonicity. A positive minimum slope indicates that the transformed retention-time axis remains strictly increasing and contains no local time axis inversions.

The minimum local slope was positive for all 134 chromatograms, confirming that all spline-based retention time transformations were strictly monotonic over the complete chromatographic range (Table S4).

Table S4. *Summary of the minimum local slopes of the spline-based retention-time transformations.*

**Statistic**	**Minimum local slope**
Number of chromatograms	134
Minimum	0.0477
25th percentile	0.6457
Median	0.8390
75th percentile	0.9103
Maximum	1.0000

S5. MATLAB code for COW optimization

This section presents the MATLAB code that uses the opt_cow() function from the PLSToolbox to optimize the parameters of the COW alignment algorithm. The optimization is performed for three different chromatographic alignment scenarios: (1) no pre-alignment (aligning ric1); (2) pre-alignment to the injection point before applying COW (aligning ric2); and (3) pre-alignment to the injection point followed by PTW adjustment before applying COW (aligning ric3). The code provides the optimal *seg* and *slack* parameters, as well as the warping functions (w1,w2, w2) and the corrected chromatograms (ricw1, ricw2, and ricw3 It is assumed that ric1, ric2, and ric3 are stored in a folder named **chromatograms** located on your desktop. The script saves all the outcomes in the same folder.

```
% Get the user's Desktop path
userDir = getenv('USERPROFILE'); % Windows
if isempty(userDir)
    userDir = getenv('HOME');    % Mac/Linux
end
desktopPath = fullfile(userDir, 'Desktop', 'chromatograms');


ref = 58;
options = [1 3 50 0.15];
optim_space = [20 200 2 50];


% Names of the chromatograms
ric_names = {'ric1', 'ric2', 'ric3'};


% Preallocate results
optim_pars_ric = zeros(length(ric_names), 4); % Assuming optim_pars returns 1x4
ricw = cell(1, numel(ric_names));
```

```
w = cell(1, numel(ric_names));

for i = 1:numel(ric_names)
   % Read chromatogram
   filename = fullfile(desktopPath, [ric_names{i}, '.csv']);
   ric = csvread(filename, 1, 1);

   % Optimize COW parameters
   [optim_pars, ~, diagnos] = optim_cow(ric, optim_space, options, ric(ref,:));

   % Store results
   optim_pars_ric(i, :) = optim_pars;
   ricw{i} = diagnos.Xw;
   w{i} = diagnos.warping(:,:,1);

   % Save corrected chromatogram and warping function
   writematrix(ricw{i}, fullfile(desktopPath, ['ricw', num2str(i), '.csv']));
   writematrix(w{i}, fullfile(desktopPath, ['w', num2str(i), '.csv']));
end

% Save optimized parameters
writematrix(optim_pars_ric, fullfile(desktopPath, 'optim_pars_ric.csv'));
```
